# Bifurcation strategies using second-generation drug-eluting stents on clinical outcomes in diabetic patients

**DOI:** 10.3389/fcvm.2022.1018802

**Published:** 2022-12-21

**Authors:** Jung-Joon Cha, Soon Jun Hong, Ju Hyeon Kim, Subin Lim, Hyung Joon Joo, Jae Hyoung Park, Cheol Woong Yu, Jeehoon Kang, Hyo-Soo Kim, Hyeon-Cheol Gwon, Woo Jung Chun, Seung-Ho Hur, Seung Hwan Han, Seung-Woon Rha, In-Ho Chae, Jin-Ok Jeong, Jung Ho Heo, Junghan Yoon, Jong-Seon Park, Myeong-Ki Hong, Joon-Hyung Doh, Kwang Soo Cha, Doo-Il Kim, Sang Yeub Lee, Kiyuk Chang, Byung-Hee Hwang, So-Yeon Choi, Myung Ho Jeong, Young Bin Song, Ki Hong Choi, Chang-Wook Nam, Bon-Kwon Koo, Do-Sun Lim

**Affiliations:** ^1^Division of Cardiology, Department of Internal Medicine, Korea University Anam Hospital, Korea University College of Medicine, Seoul, South Korea; ^2^Department of Internal Medicine and Cardiovascular Center, Seoul National University Hospital, Seoul, South Korea; ^3^Department of Internal Medicine, Samsung Medical Center, Sungkyunkwan University School of Medicine, Seoul, South Korea; ^4^Department of Internal Medicine, Samsung Changwon Hospital, Sungkyunkwan University School of Medicine, Changwon, South Korea; ^5^Department of Internal Medicine, Keimyung University Dongsan Medical Center, Daegu, South Korea; ^6^Department of Internal Medicine, Gachon University Gil Hospital, Incheon, South Korea; ^7^Department of Internal Medicine, Korea University Guro Hospital, Seoul, South Korea; ^8^Department of Internal Medicine, Seoul National University Bundang Hospital, Seongnam, South Korea; ^9^Department of Medicine, Chungnam National University Hospital, Daejeon, South Korea; ^10^Department of Internal Medicine, Kosin University Gospel Hospital, Kosin University College of Medicine, Pusan, South Korea; ^11^Department of Internal Medicine, Wonju Severance Christian Hospital, Yonsei University Wonju College of Medicine, Wonju, South Korea; ^12^Department of Internal Medicine, Yeungnam University Medical Center, Daegu, South Korea; ^13^Department of Internal Medicine, Severance Cardiovascular Hospital, Yonsei University College of Medicine, Seoul, South Korea; ^14^Department of Internal Medicine, Inje University Ilsan Paik Hospital, Ilsan, South Korea; ^15^Department of Internal Medicine, Pusan National University Hospital, Pusan, South Korea; ^16^Department of Internal Medicine, Inje University Haeundae Paik Hospital, Pusan, South Korea; ^17^Department of Cardiology, Chung-Ang University, College of Medicine Heart and Brain Hospital, Chung-Ang University Gwangmyeong Hospital, Gwangmyeong, South Korea; ^18^Department of Internal Medicine, Seoul St. Mary's Hospital, The Catholic University of Korea, Seoul, South Korea; ^19^Department of Internal Medicine, St. Paul's Hospital, The Catholic University of Korea, Seoul, South Korea; ^20^Department of Internal Medicine, Ajou University Hospital, Suwon, South Korea; ^21^Department of Internal Medicine, Chonnam National University Hospital, Gwangju, South Korea

**Keywords:** coronary bifurcation angioplasty, diabetes mellitus, stent strategy, second-generation drug-eluting stent, clinical outcome, percutaneous coronary intervention (complex PCI)

## Abstract

**Background:**

Diabetes mellitus (DM) is a critical risk factor for the pathogenesis and progression of coronary artery disease, with a higher prevalence of complex coronary artery disease, including bifurcation lesions. This study aimed to elucidate the optimal stenting strategy for coronary bifurcation lesions in patients with DM.

**Methods:**

A total of 905 patients with DM and bifurcation lesions treated with second-generation drug-eluting stents (DES) from a multicenter retrospective patient cohort were analyzed. The primary outcome was the 5-year incidence of target lesion failure (TLF), which was defined as a composite of cardiac death, target vessel myocardial infarction, and target lesion revascularization.

**Results:**

Among all patients with DM with significant bifurcation lesions, 729 (80.6%) and 176 (19.4%) were treated with one- and two-stent strategies, respectively. TLF incidence differed according to the stenting strategy during the mean follow-up of 42 ± 20 months. Among the stent strategies, T- and V-stents were associated with a higher TLF incidence than one-stent strategy (24.0 vs. 7.3%, *p* < 0.001), whereas no difference was observed in TLF between the one-stent strategy and crush or culotte technique (7.3 vs. 5.9%, *p* = 0.645). The T- or V-stent technique was an independent predictor of TLF in multivariate analysis (hazard ratio, 3.592; 95% confidence interval, 2.117–6.095; *p* < 0.001). Chronic kidney disease, reduced left ventricular ejection fraction, and left main bifurcation were independent predictors of TLF in patients with DM.

**Conclusion:**

T- or V-stenting in patients with DM resulted in increased cardiovascular events after second-generation DES implantation.

**Clinical trial registration:**

https://clinicaltrials.gov/ct2/show/NCT03068494?term=03068494&draw=2&rank=1, identifier: NCT03068494.

## 1. Introduction

Diabetes mellitus (DM) is an independent predictor of long-term death, myocardial infarction, and revascularization in patients undergoing percutaneous coronary intervention (PCI) (1–3). This may be due to impaired endothelial function caused by DM, which promotes a pro-inflammatory vasoconstrictive state and prompts arterial atherothrombosis (4, 5). Thus, newer-generation drug-eluting stents (DES) are recommended for patients with DM undergoing PCI rather than bare-metal stent or early-generation DESs (6). However, despite stent technology and strategy improvements, patients with DM after PCI presented poorer clinical outcomes than patients without DM (7, 8).

A new stent technology achieving high therapeutic drug concentrations in the arterial tissue using a reservoir recently presented better clinical outcomes in patients with DM after PCI (9). These results may lead to identifying DM-specific treatments. However, little is known concerning the optimal stent strategy for complex PCI cases, such as coronary bifurcation diseases associated with atherosclerosis progression and thrombosis due to higher endothelial shear stress, especially in patients with DM (10, 11). In addition, clinical outcomes of stent strategies for coronary bifurcation lesions in patients with DM using second-generation DES have not been fully elucidated.

Thus, this study aimed to investigate the impact of stenting strategies on clinical outcomes in patients with DM and coronary bifurcation lesions using second-generation DES.

## 2. Methods

### 2.1. Study population

This retrospective study cohort was based on the coronary bifurcation stent III registry (NCT03068494) and consisted of 2,648 patients treated between January 2010 and December 2014 in 21 Korean tertiary hospitals. The design and detailed description of the registry have been previously reported (12). The coronary bifurcation stent III registry is a real-world registry of second-generation DES use, and from the registry, patients with DM (*N* = 905) were included in this study. The inclusion criteria were age >19 years and main vessel (MV) diameter ≥2.5 mm and side branch (SB) diameter ≥2.3 mm, confirmed using core laboratory quantitative coronary angiography analysis. Patients who experienced cardiogenic shock or cardiopulmonary resuscitation during hospitalization, had protected left main disease, or had severe left ventricular systolic dysfunction (ejection fraction <30%) were excluded from the registry. The institutional review board of each hospital approved the study protocol, which was conducted in accordance with the principles of the Declaration of Helsinki. Each institutional review board waived the requirement for informed consent due to the retrospective nature of the study.

### 2.2. Percutaneous coronary bifurcation intervention

Index PCI was performed according to the relevant standard guidelines during each procedure. Before PCI, all patients received loading doses of antiplatelet medications (aspirin 300 mg and P2Y12 inhibitors [clopidogrel 300–600 mg, prasugrel 60 mg, or ticagrelor 180 mg]) unless they had previously received antiplatelet therapy. An activated clotting time of 250–300 s was maintained during PCI using low-molecular-weight or unfractionated heparin. The PCI strategy, including stent strategy, proximal optimization technique (POT) or re-POT, access site, DES type, glycoprotein IIb/IIIa inhibitor use, and intravascular imaging or invasive physiological assessments, was based on the operator's discretion. In addition, the duration of dual antiplatelet therapy and DM medication was at the operator's discretion.

### 2.3. Data collection and quantitative coronary angiography analysis

Patient information, including demographics; medication; and laboratory, angiographic, and procedural data, was collected for analysis through a web-based reporting system. Follow-up clinical outcomes were obtained from electronic medical records of the outpatient clinic. For the quantitative coronary angiography (QCA) analysis, an angiographic core laboratory (Heart Vascular Stroke Institute, Samsung Medical Center, Seoul, South Korea) with a validated automated edge-detection system (Centricity CA 1000; GE, Waukesha, WI, USA) reviewed and analyzed all baseline and procedural coronary angiograms. QCA analysis was performed pre- and post-procedure, bifurcation angle (the angle between the distal MV and the SB at its origin, measured using the angiographic projection with the widest separation of both branches), minimum lumen diameter, reference vessel diameter, and lesion length for each vessel were measured. In addition, percent diameter stenosis (100 × [reference vessel diameter/minimum lumen diameter]/reference vessel diameter) for each vessel was determined.

### 2.4. Primary and secondary outcomes

The primary outcome was the 5-year incidence of target lesion failure (TLF), defined as the composite of cardiac death, target vessel myocardial infarction (TVMI), and target lesion revascularization (TLR). The secondary outcomes were the individual components of the primary outcome. An independent clinical event adjudication committee composed of independent interventional cardiology experts who had not participated in patient enrollment verified all the clinical events. Deaths were of cardiac cause unless an undisputed non-cardiac cause could be established. TVMI was myocardial infarction with evidence of an elevated creatine kinase-myocardial band or a troponin level higher than the standard upper limit with concomitant ischemic symptoms or electrocardiography findings indicative of ischemia in the vascular territory of the previously treated target vessels. TLR was repeat PCI of the lesion within 5 mm of the stent deployment.

### 2.5. Statistical analysis

Continuous variables are presented as mean ± standard deviation and were compared using the Student's *t*-test for parametric data and the Mann–Whitney test for non-parametric data. Categorical variables are presented as numbers (percentages) and were compared using the Chi-squared test or Fisher's exact test. The cumulative incidences of clinical events are presented as Kaplan–Meier estimates and compared using a log-rank test. Patients were censored at 5 years (1,825 days) or when events occurred. Hazard ratios (HRs) and 95% confidence intervals (CIs) were calculated using the Cox proportional hazards models. In multivariable models, variables with *p*-values <0.10 in the univariate analysis were included in the multivariate analysis using backward elimination and multivariable Cox regression to determine the independent predictors of clinical events. After univariate analysis, adjusted HR was obtained from Cox regression based on taking insulin for DM, chronic kidney disease, preserved left ventricular ejection fraction (LVEF; ≥50%), left main (LM) bifurcation, stent strategy, post-procedural distal minimal lumen diameter of the SB, and final kissing balloon (FKB) inflation. All probability values were two-sided, and *p*-values <0.05 were significant. Propensity scores were estimated using a non-parsimonious multiple logistic regression model for stent strategy. Age, sex, initial presentation, hypertension, taking insulin for DM, dyslipidemia, current smoking status, chronic kidney disease, previous myocardial infarction, previous percutaneous coronary intervention, left ventricular ejection fraction <50%, transradial approach, use of intravascular ultrasound, left main bifurcation, and true bifurcation were selected to estimate the propensity score. A local optimal algorithm using the caliper method was used to develop propensity score-matched pairs without replacement (2:1 matching). To ensure that poorly fitting matches were excluded, a matching caliper of 0.2 SDs from the estimated propensity score logit was enforced using the MatchIt package in R Core Team (2015). R: Language and environment for statistical computing (version 3.6.0, R Foundation for Statistical Computing, Vienna, Austria; https://www.R-project.org/). SPSS version 25.0 software (IBM Corp., Armonk, NY, USA) was used to analyze the results.

## 3. Results

### 3.1. Patients' baseline characteristics

A total of 905 patients with DM and significant bifurcation lesions were enrolled in the study: 729 (80.6%) were treated with a one-stent strategy, and 176 (19.4%) were treated with a two-stent strategy. The baseline clinical and procedural characteristics of all patients with DM according to the stenting strategy are presented in Table 1. There was no significant difference between the two-stent and one-stent groups except in the prevalence of insulin use (14.2 vs. 8.2%, *p* = 0.022). The two-stent group had a higher prevalence of intravascular ultrasound use (54.5 vs. 35.8%, *p* < 0.001), LM bifurcation (53.4 vs. 35.9%, *p* < 0.001), true bifurcation lesions (78.4 vs. 38.8%, *p* < 0.001), and FKB inflation (87.5 vs. 17.3%, *p* < 0.001) than the one-stent group. The transradial approach was used less often in the two-stent group than in the one-stent group (38.1 vs. 61.2%, *p* < 0.001). The QCA results are presented in Supplementary Table 1.

**Table 1:** Patients' baseline clinical and procedural characteristics.

	**Total**	**One-stent**	**Two-stent**	***P*-value**
	**(*N* = 905)**	**(*n* = 729)**	**(*n* = 176)**	
Age, years	65.3 ± 10.0	65.0 ± 10.2	66.2 ± 9.4	0.147
Male sex	653 (72.2%)	533 (73.1%)	120 (68.2%)	0.224
**Initial presentation**				0.861
Stable angina	370 (40.9%)	295 (40.5%)	75 (42.6%)	
NSTE-ACS	458 (50.6%)	371 (50.9%)	87 (49.4%)	
STEMI	77 (8.5%)	63 (8.6%)	14 (8.0%)	
Hypertension	592 (65.4%)	481 (66.0%)	111 (63.1%)	0.522
DM taking insulin	85 (9.4%)	60 (8.2%)	25 (14.2%)	0.022
Dyslipidemia	362 (40.0%)	297 (40.7%)	65 (36.9%)	0.401
Current smoking status	241 (26.6%)	199 (27.3%)	42 (23.9%)	0.407
CKD	72 (8.0%)	56 (7.7%)	16 (9.1%)	0.642
Previous MI	43 (4.8%)	34 (4.7%)	9 (5.1%)	0.957
Previous PCI	145 (16.0%)	113 (15.5%)	32 (18.2%)	0.450
LVEF <50%	138 (15.2%)	105 (14.4%)	33 (18.8%)	0.186
Transradial approach	513 (56.7%)	446 (61.2%)	67 (38.1%)	< 0.001
IVUS use	357 (39.4%)	261 (35.8%)	96 (54.5%)	< 0.001
LM bifurcation	356 (39.3%)	262 (35.9%)	94 (53.4%)	< 0.001
True bifurcation	421 (46.5%)	283 (38.8%)	138 (78.4%)	< 0.001
**Stent strategy**				
Simple crossover	562 (62.1%)	562 (77.1%)	-	
One-stent with SB balloon	167 (18.5%)	167 (22.9%)	-	
Crush	81 (9.0%)	-	81 (46.0%)	
T (or TAP)	55 (6.1%)	-	55 (31.2%)	
Culotte	16 (1.8%)	-	16 (9.1%)	
V (or kissing)	20 (2.2%)	-	20 (11.4%)	
Other	4 (0.4%)	-	4 (2.3%)	
**DES type**				0.287
Everolimus eluting stent	446 (49.3%)	348 (47.7%)	98 (55.7%)	
Zotarolimus eluting stent	249 (27.5%)	207 (28.4%)	42 (23.9%)	
Biolimus eluting stent	163 (18.0%)	134 (18.4%)	29 (16.5%)	
Others	47 (5.2%)	40 (5.5%)	7 (4.0%)	
Kissing balloon inflation	280 (30.9%)	126 (17.3%)	154 (87.5%)	<0.001
Proximal optimization technique	283 (31.3%)	228 (31.3%)	55 (31.2%)	>0.999
MV success	899 (99.3%)	723 (99.2%)	176 (100.0%)	0.490
SB success	649 (71.7%)	476 (65.3%)	173 (98.3%)	<0.001

CKD, chronic kidney disease; DM, diabetes mellitus; IVUS, intravascular ultrasound; LM, left main; LVEF, left ventricular ejection fraction; MI, myocardial infarction; MV, main vessel; NSTE-ACS, non-ST elevation–acute coronary syndrome; PCI, percutaneous intervention; SB, side branch; STEMI, ST-segment elevation myocardial infarction; TAP, T, and small protrusions.

### 3.2. Outcomes and TLF predictors

**Figure 1:**
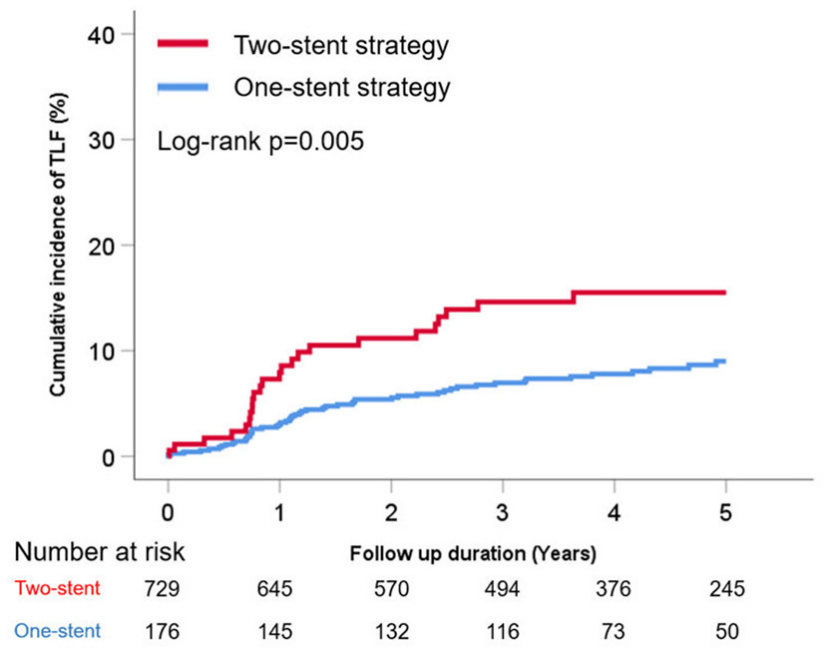
Cumulative incidence of target lesion failure (TLF) of one- vs. two-stent strategy for treating coronary bifurcation lesions in patients with diabetes mellitus.

**Figure 2:**
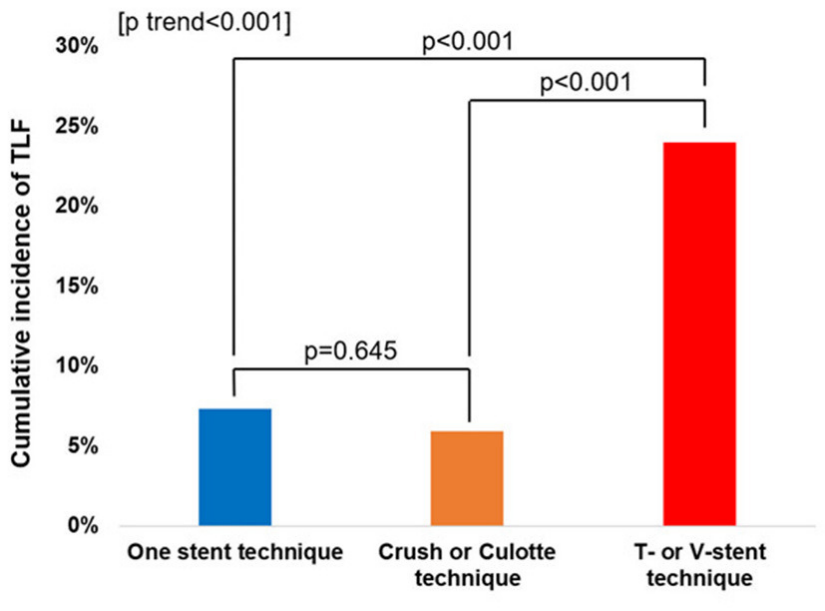
Incidence of target lesion failure (TLF) for one-stent, T-stent, V-stent, and crush or culotte techniques.

**Figure 3:**
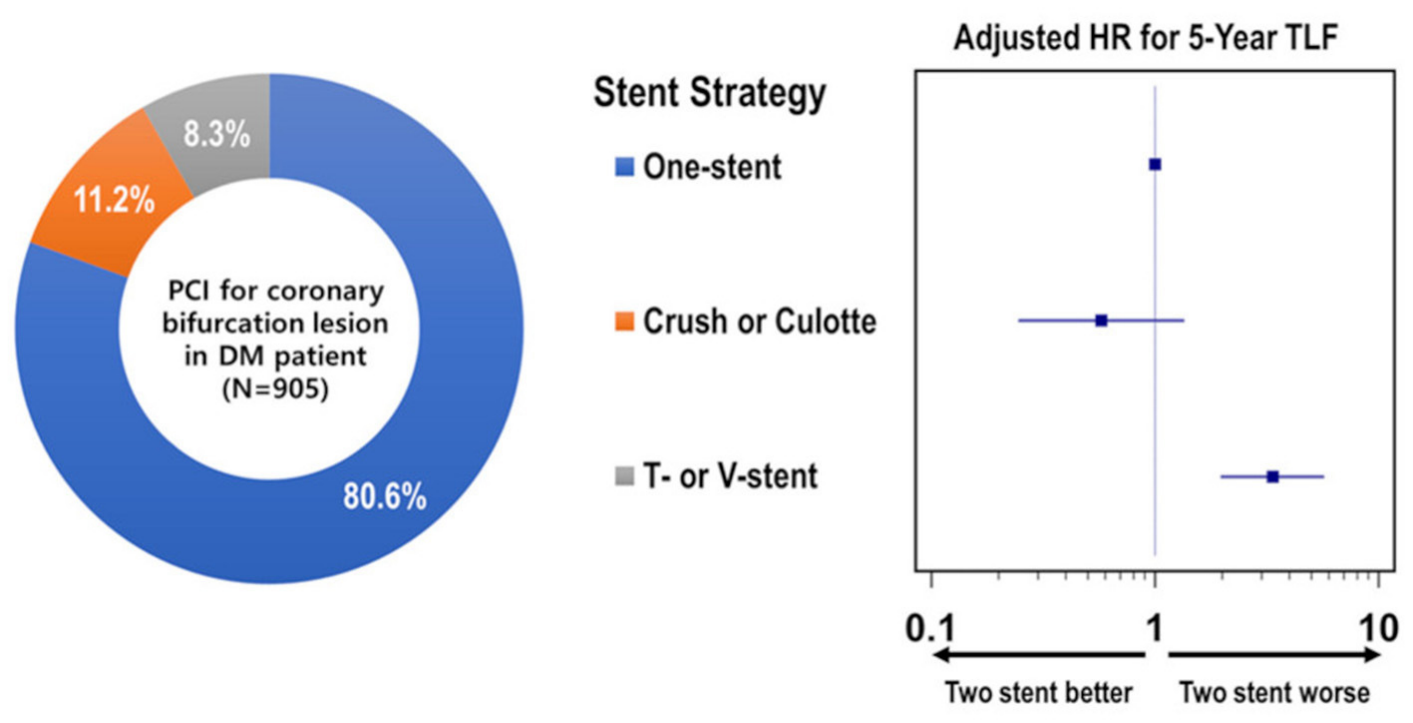
Distribution and individual risk of stent strategy for coronary bifurcation lesions in patients with diabetes mellitus.

The cumulative TLF incidence was higher in patients treated with two- than one-stent strategy (13.6 vs. 7.3%, *p* = 0.005; Figure 1). Notably, at a mean follow-up of 42 ± 20 months, there was a significant difference in TLF incidence based on the stenting strategy (Supplementary Figure 1; *p* for trend <0.001), and the T- or V-stent strategy demonstrated a higher TLF incidence than the one-stent strategy (24.0 vs. 7.3%, *p* < 0.001). However, the crush or culotte technique and the one-stent strategy presented similar outcomes (5.9 vs. 7.3%, *p* = 0.645; Figure 2). In the Cox multivariate analysis, the T- or V-stent technique remained significantly associated with TLF (HR, 3.349; 95% CI, 1.960–5.721; *p* < 0.001), mainly driven by TLR (HR, 4.688; 95% CI, 2.478–8.869; *p* < 0.001), with no significant differences in cardiac death or TVMI. Meanwhile, the crush and culotte techniques did not have significantly different clinical outcomes (Figure 3). In addition, chronic kidney disease (CKD; HR, 3.071; 95% CI, 1.728–5.456; *p* < 0.001), reduced LVEF (HR, 2.436; 95% CI, 1.478–4.017; *p* < 0.001), and LM bifurcation (HR, 2.030; 95% CI, 1.290–3.195; *p* = 0.002) during follow-up were independent predictors of TLF in patients with DM after multivariate adjustment (Table 2). After propensity score matching (Supplementary Table 2), although there was no TLF difference between the one- and two-stent groups (11.8 vs. 8.9%, *p* = 0.334; Supplementary Figure 2A), the T- or V-stent technique had a higher TLF incidence than the others (Supplementary Figure 2B; *p* for trend = 0.025). In addition, the T- or V-stent technique remained significantly associated with TLF (HR, 2.269; 95% CI, 1.123–4.584; *p* = 0.022) in the propensity score matching.

**Table 2:** Independent predictors of 5-year target lesion failure in DM patients treated for coronary bifurcation lesion with stent implantation.

**All patients with DM (*N* = 905)**	**Crude HR**	**Final model, stepwise backward elimination (HR and 95% CI)**	***P*-value**
Use of insulin	2.143	1.772 (0.951–3.301)	0.074
Chronic kidney disease	3.793	3.034 (1.710–5.385)	<0.001
Preserved LVEF (≥50%)	0.405	0.419 (0.254–0.690)	0.001
Radial artery approach	0.633	-	-
Left main bifurcation	1.975	1.932 (1.233–3.028)	0.004
T- or V-stent technique	1.330	3.592 (2.117–6.095)	<0.001
Kissing balloon inflation	0.470	-	-
Post- procedural distal MLD of SB	0.410	-	-

CI, confidence interval; DM, diabetes mellitus; HR, hazard ratio; LVEF, left ventricular ejection fraction; MLD, minimal lumen diameter; SB, side branch.

## 4. Discussion

This study investigated the impact of stenting strategies on the clinical outcomes of patients with DM who were treated for coronary bifurcation lesions. This study revealed that the one-stent strategy for coronary bifurcation lesions presented a lower TLF incidence than the two-stent strategy, in patients with DM. The T- or V-stent technique also revealed a higher TLF incidence, mainly driven by TLR, than the one-stent strategy and crush or culotte technique. Furthermore, except for the T- or V-stent techniques, there was no difference in TLF incidence between the one- and two-stent strategies. Additionally, using the T- or V-stent technique in patients with DM undergoing PCI for coronary bifurcation lesions was an independent predictor of TLF in the multivariate analysis. CKD, reduced LVEF, and left main bifurcation lesions were independent TLF predictors.

Patients with DM are at high risk of progressive atherosclerosis regarding coronary plaque rupture and neointimal proliferation, which leads to an increased incidence of adverse clinical outcomes (4, 13). Moreover, PCI with coronary bifurcation lesions presented a higher incidence of MI, thrombosis, and revascularization than PCI with simple coronary lesions (14). A plausible explanation for the adverse prognosis after coronary bifurcation PCI is the unique local flow pattern that increases the endothelial shear stress and affects plaque development. According to our previous study, the 5-year TLF incidence was 7.8% in patients with and without DM who underwent PCI for coronary bifurcation lesions (15). The 5-year incidence rates of TLF for the one- and two-stent strategies were 7.6 and 12.1%, respectively. In this study, the 5-year incidence rate of TLF in patients with DM was 8.5%. This difference in TLF rate between the total and DM populations was primarily due to the different events in the two-stent strategy (12.1 vs. 13.6%, respectively).

To date, no randomized controlled trial or large-scale observational study has reported the clinical outcomes of coronary bifurcation lesions in patients with DM regarding stent strategy in the second-generation drug-eluting stent era. In this study, the T- or T and small protrusion (TAP) technique and V- or simultaneous kissing stenting revealed a higher TLF incidence than the other techniques and were independent predictors of TLF in multivariable analysis. To our knowledge, this is the first study to reveal that different stent strategies in the two-stent technique may be associated with clinical outcomes in patients with coronary bifurcation lesions. Consistent with this study, a meta-analysis of various randomized controlled trials on coronary bifurcation stent strategies reported that the TLR rate of the T-stent or TAP technique was higher than that of other techniques (16). In the DEFINITION II trial, which included 35% of patients with DM, the double kissing crush technique had a 1-year TLR rate, superior to that of the provisional stent group (14.5 %) using the T-stent or TAP technique for bailout stenting (17).

A possible explanation for these results is the association between DM and shear stress at the bifurcation level (18). The T-stent technique is commonly used when the SB is compromised during provisional stenting; however, it has the inherent risk of suboptimal SB ostium coverage, which may lead to restenosis (19). The V-stent technique has a similar limitation regarding the suboptimal coverage of the SB ostium. The TAP stent technique has a lower risk of missing the SB ostium (19); however, it creates a metallic neocarina, where a significant portion of the unappositioned stent remains in the vessel. In addition, the post-procedural minimal lumen diameter of the main branch ostium was an independent TLF predictor. Theoretically, metallic neocarina may risk narrowing the main branch ostium. A recent study suggested obtaining the maximal diameter of the main vessel stent in PCI for a non-left main bifurcation lesion (12). In our previous study, DM was an independent TLF predictor in non-LM lesions but not in LM bifurcation lesions (12). DM is a potential factor in atherosclerosis progression and neointimal proliferation (4) and a vasoconstrictive endothelial response along with an inflammatory and prothrombotic milieu in DM. A synergistic relationship with shear stress at the bifurcation level may lead to a worse prognosis (13, 18, 20). In this study's subgroup analysis, except for the T-stent, TAP stent, and V-stent techniques, there was no significant difference in the TLF between the one- and two-stent strategies. These observations suggest that accurate stent deployment and optimization of the bifurcation carina site could reduce TLF incidence, especially in the very high-risk population with DM and bifurcation CAD (19).

In addition, CKD, reduced LVEF, and LM bifurcation were independent TLF predictors in the diabetic population. CKD is associated with a poor prognosis due to its strong correlation with various risk factors, such as hypertension, DM, and dyslipidemia, which could be a cause or consequence (21). In addition, DM is an extremely high-risk factor for patients referred for treatment of LM bifurcation. Our study also emphasizes that PCI for LM bifurcation is an independent TLF predictor in patients with DM, even in the second-generation DES era, consistent with that reported in previous studies (12, 15, 22, 23).

This study had several limitations. First, its inherent limitation is the nature of the observational registry. The multivariate adjustment was performed; nonetheless, potential bias due to unmeasured variables or confounding factors, such as body mass index, serum glucose level, and mean blood pressure, could not be excluded. Second, the range of baseline and follow-up glycemic levels of these patients could have influenced our results, and insulin use revealed a borderline significant risk of TLF (adjusted HR, 1.811; *p* = 0.061), which suggests that poor DM control may affect clinical outcomes, highlighting the need for further research. Finally, treatment strategy, intravascular imaging, stent type, and concomitant medication use were based on the physician's preferences. In this context, FKB inflation was relatively poorly performed in patients treated with the two-stent strategy, and the rate of POT was relatively low. However, our study has analyzed the largest real-world PCI dataset for bifurcation lesions in patients with DM.

## 5. Conclusion

T- or V-stenting in patients with DM showed increased cardiovascular events after second-generation DES implantation compared with one- or other two-stent strategies.

## Data availability statement

The original contributions presented in the study are included in the article/Supplementary material, further inquiries can be directed to the corresponding author.

## Ethics statement

The studies involving human participants were reviewed and approved by the Institutional Review Board of each hospital including Korea University Anam Hospital and conducted in accordance with the principles of the Declaration of Helsinki. Written informed consent for participation was not required for this study in accordance with the national legislation and the institutional requirements.

## Author contributions

All authors listed have made a substantial, direct, and intellectual contribution to the work and approved it for publication.
