# Supplementary material for: Bifurcation strategies using second-generation drug-eluting stents on clinical outcomes in diabetic patients
Source: Front Cardiovasc Med. 2022 Dec 21;9:1018802. doi: 10.3389/fcvm.2022.1018802 (PMC9811589; doi:10.3389/fcvm.2022.1018802)
Supplement: Supplementary file 1 [file Data_Sheet_1.docx]

**Supplementary material**

**Supplemental Figure 1.** Cumulative incidence of target lesion failure (TLF) by individual stent strategy for coronary bifurcation lesions in patients with diabetes mellitus


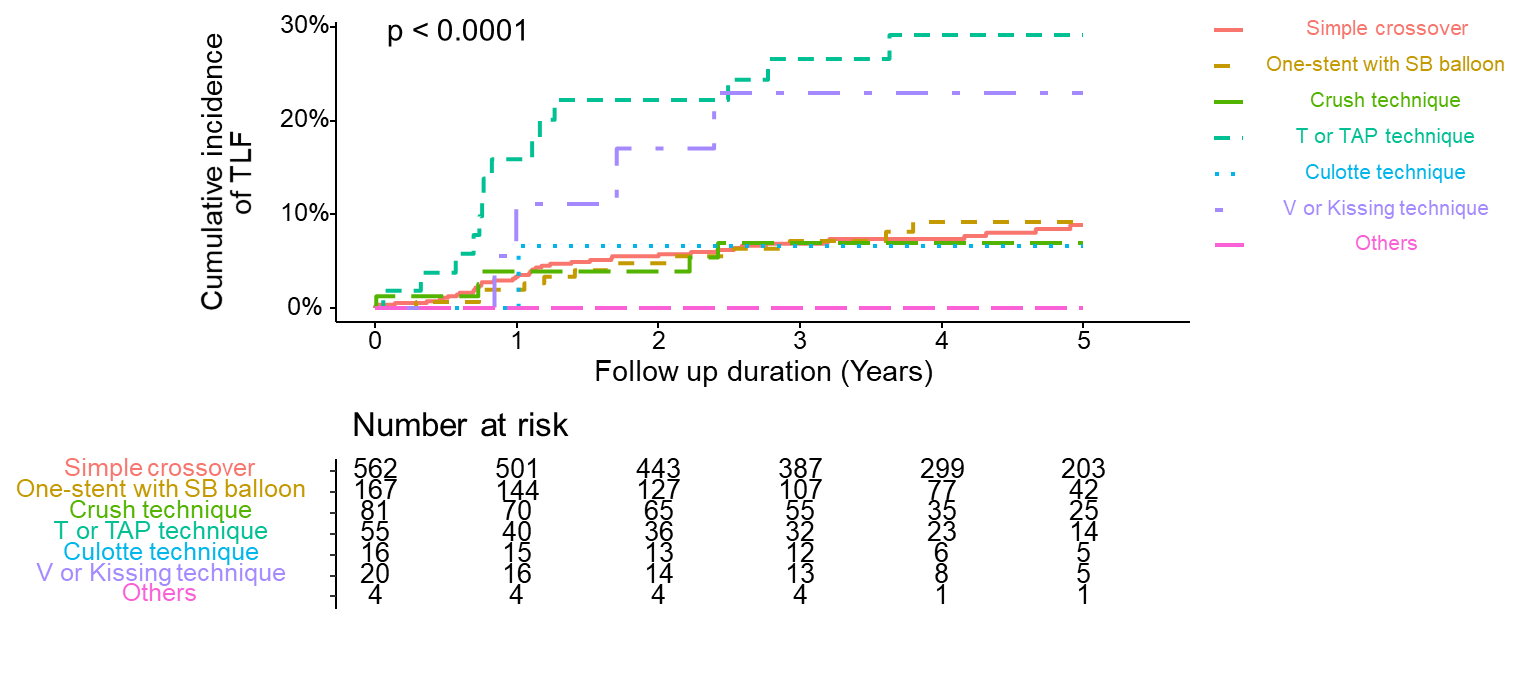


**Supplemental Figure 2.** After propensity score matching, the cumulative incidence of target lesion failure (TLF) according to (A) one-stent vs. two-stent strategy, (B) one-stent vs. crush or culotte vs. T- or V-stent technique.


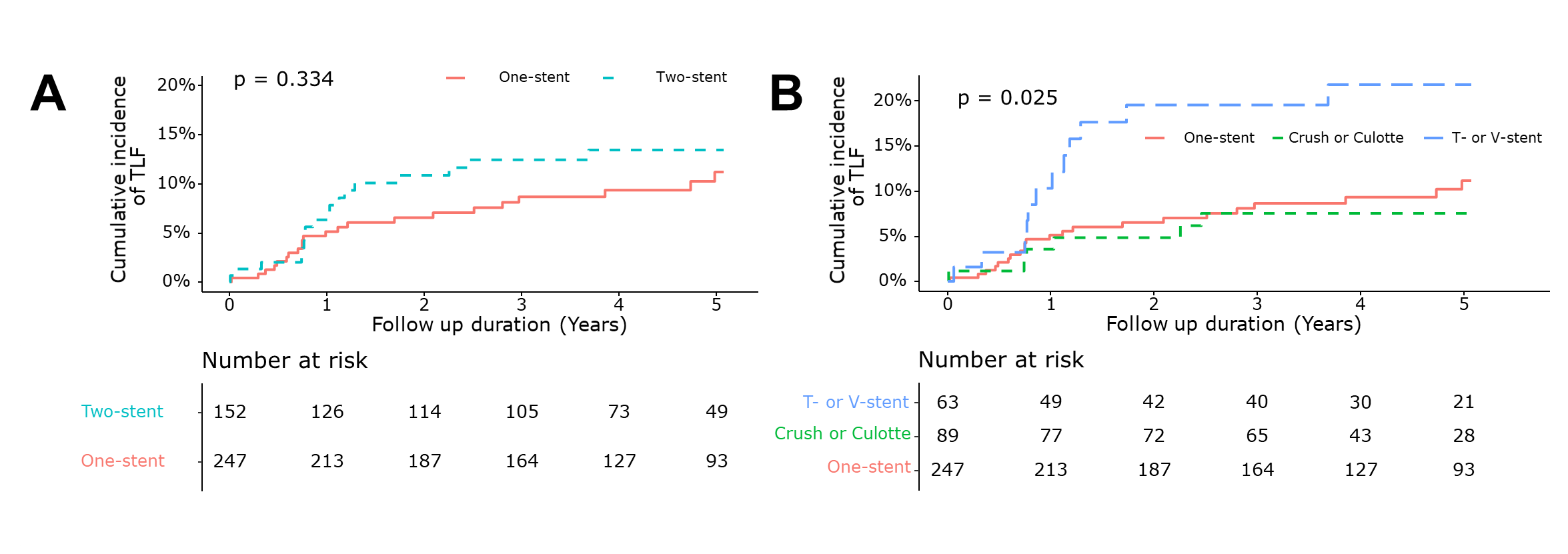


**Supplemental Table 1.** Quantitative coronary angiography analysis by stent approach

|  | **Total** | **One-stent** | **Two-stent** | **p value** |
| --- | --- | --- | --- | --- |
|  | **(N=905)** | **(n=729)** | **(n=176)** |  |
| **Pre-procedural measurement** |  |  |  |  |
| **PVRD** | 3.6 ± 0.6 | 3.6 ± 0.6 | 3.7 ± 0.7 | 0.047 |
| **MBRD** | 2.8 ± 0.4 | 2.8 ± 0.4 | 2.8 ± 0.5 | 0.543 |
| **SBRD** | 2.6 ± 0.4 | 2.6 ± 0.4 | 2.6 ± 0.5 | 0.148 |
| **PVMLD** | 1.8 ± 1.1 | 1.8 ± 1.1 | 1.9 ± 1.0 | 0.102 |
| **MBostialMLD** | 1.2 ± 0.7 | 1.2 ± 0.7 | 1.2 ± 0.7 | 0.635 |
| **MBdistalMLD** | 1.8 ± 0.9 | 1.8 ± 0.9 | 1.7 ± 0.9 | 0.328 |
| **SBostialMLD** | 1.6 ± 0.8 | 1.7 ± 0.8 | 1.0 ± 0.6 | < 0.001 |
| **SBdistalMLD** | 2.0 ± 0.8 | 2.1 ± 0.7 | 1.6 ± 0.8 | < 0.001 |
| **MVD** | 3.2 ± 0.5 | 3.2 ± 0.5 | 3.2 ± 0.5 | 0.311 |
| **MVMLD** | 0.9 ± 0.5 | 0.8 ± 0.4 | 1.0 ± 0.6 | 0.003 |
| **SBMLD** | 1.5 ± 0.8 | 1.6 ± 0.8 | 0.9 ± 0.5 | < 0.001 |
| **MV length** | 19.2 ± 10.5 | 19.6 ± 10.2 | 17.8 ± 11.5 | 0.058 |
| **SB length** | 5.8 ± 7.2 | 4.3 ± 6.3 | 11.6 ± 7.9 | <0.001 |
| **Post-procedural measurement** |  |  |  |  |
| **PVRD** | 3.6 ± 0.6 | 3.6 ± 0.6 | 3.7 ± 0.6 | 0.014 |
| **MBRD** | 2.9 ± 0.4 | 2.9 ± 0.4 | 2.9 ± 0.5 | 0.789 |
| **SBRD** | 2.6 ± 0.4 | 2.6 ± 0.4 | 2.7 ± 0.4 | 0.021 |
| **PVMLD** | 3.2 ± 0.6 | 3.2 ± 0.6 | 3.4 ± 0.6 | < 0.001 |
| **MBostialMLD** | 2.9 ± 0.5 | 2.9 ± 0.5 | 2.9 ± 0.4 | 0.530 |
| **MBdistalMLD** | 2.9 ± 0.5 | 2.9 ± 0.5 | 2.8 ± 0.4 | 0.629 |
| **SBosMLD** | 1.8 ± 0.8 | 1.6 ± 0.8 | 2.4 ± 0.5 | < 0.001 |
| **SBdistalMLD** | 2.2 ± 0.6 | 2.1 ± 0.7 | 2.6 ± 0.4 | < 0.001 |
| **MVD** | 3.3 ± 0.5 | 3.2 ± 0.5 | 3.3 ± 0.5 | 0.140 |
| **MVMLD** | 2.7 ± 0.5 | 2.7 ± 0.5 | 2.7 ± 0.4 | 0.841 |
| **SBMLD** | 1.7 ± 0.8 | 1.6 ± 0.8 | 2.4 ± 0.5 | < 0.001 |

MB, main branch; MLD, minimal lumen diameter; PV, parent vessel; RD, reference diameter; SB, side branch

**Supplemental Table 2.** Patients’ baseline clinical and procedural characteristics after propensity score matching

|  | **One-stent** | **Two-stent** | ***P*-value** |
| --- | --- | --- | --- |
|  | **(N=247)** | **(N=152)** |  |
| **Age, years** | 65.7 ± 10.5 | 65.9 ± 9.3 | 0.817 |
| **Male sex** | 176 (71.3%) | 104 (68.4%) | 0.625 |
| **Initial presentation** |  |  | 0.861 |
| **Stable angina** | 105 (42.5%) | 63 (41.4%) |  |
| **NSTE-ACS** | 116 (47.0%) | 75 (49.3%) |  |
| **STEMI** | 26 (10.5%) | 14 ( 9.2%) |  |
| **Hypertension** | 162 (65.6%) | 100 (65.8%) | 1.000 |
| **DM taking insulin** | 28 (11.3%) | 19 (12.5%) | 0.849 |
| **Dyslipidemia** | 96 (38.9%) | 59 (38.8%) | 1.000 |
| **Current smoking status** | 63 (25.5%) | 36 (23.7%) | 0.772 |
| **CKD** | 22 ( 8.9%) | 14 ( 9.2%) | 1.000 |
| **Previous MI** | 11 ( 4.5%) | 7 ( 4.6%) | 1.000 |
| **Previous PCI** | 43 (17.4%) | 28 (18.4%) | 0.903 |
| **LVEF <50%** | 44 (17.8%) | 26 (17.1%) | 0.964 |
| **Transradial approach** | 107 (43.3%) | 64 (42.1%) | 0.893 |
| **IVUS use** | 119 (48.2%) | 75 (49.3%) | 0.902 |
| **LM bifurcation** | 105 (42.5%) | 75 (49.3%) | 0.219 |
| **True bifurcation** | 167 (67.6%) | 114 (75.0%) | 0.145 |
